# Supplementary figures and images for: Behavioural patterns of vocal greeting production in four primate species
Source: R Soc Open Sci. 2019 Apr 24;6(4):182181. doi: 10.1098/rsos.182181 (PMC6502363; doi:10.1098/rsos.182181)

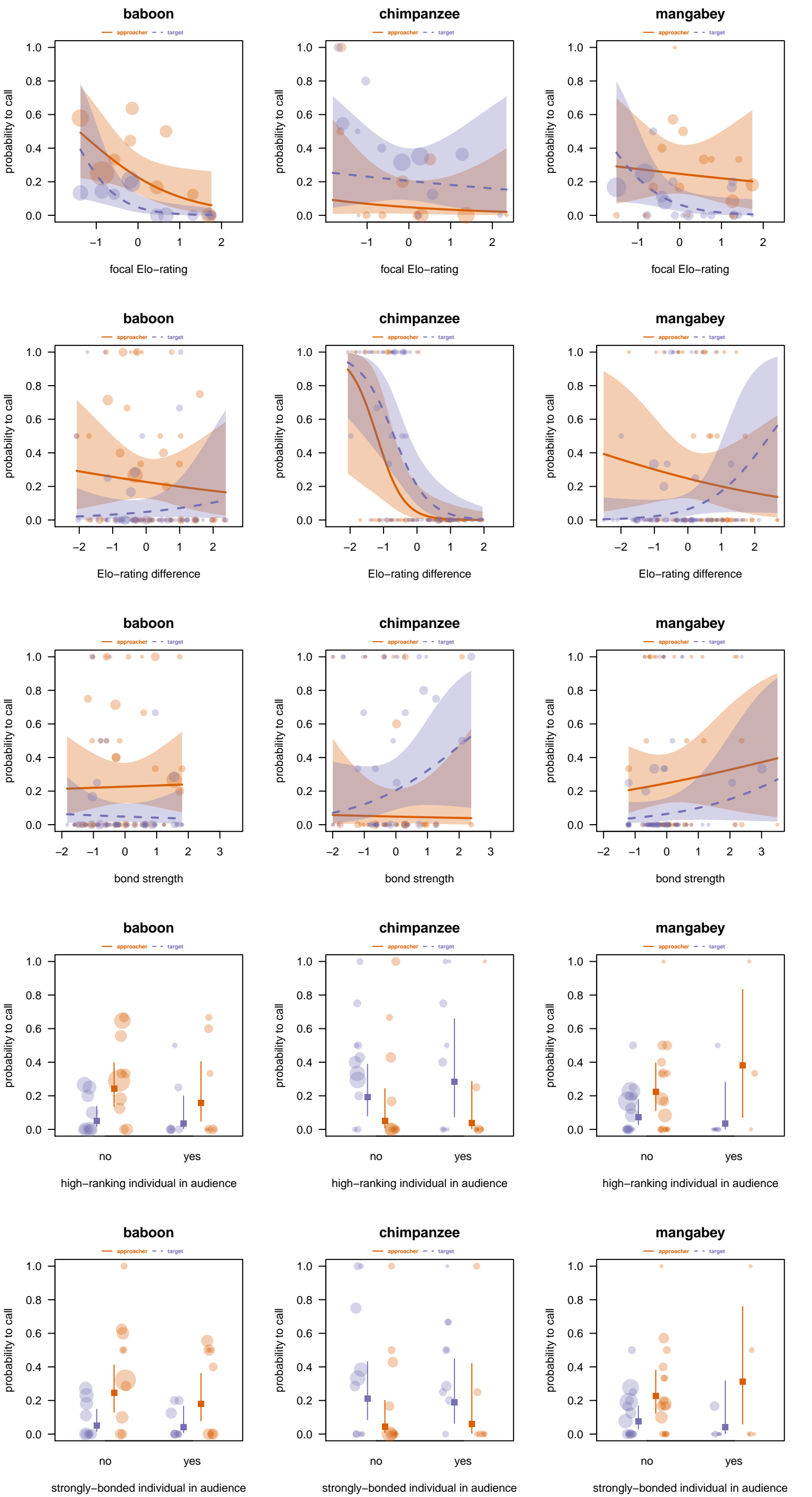

Supplement: Fig S1 [file rsos182181supp1.pdf]
